# Supplementary material for: Prediction of air quality in Sydney, Australia as a function of forest fire load and weather using Bayesian statistics
Source: PLoS One. 2022 Aug 24;17(8):e0272774. doi: 10.1371/journal.pone.0272774 (PMC9401134; doi:10.1371/journal.pone.0272774)
Supplement: S1 Appendix — (PDF) [file pone.0272774.s001.pdf]

# S1 Appendix

This appendix contains effects plots for the maximum Sydney $PM_{2.5}$ model and tile plots with predictions for both the mean and maximum $PM_{2.5}$ models with different thresholds to those in the main text. The plots are discussed in the results and discussion sections in the main text.

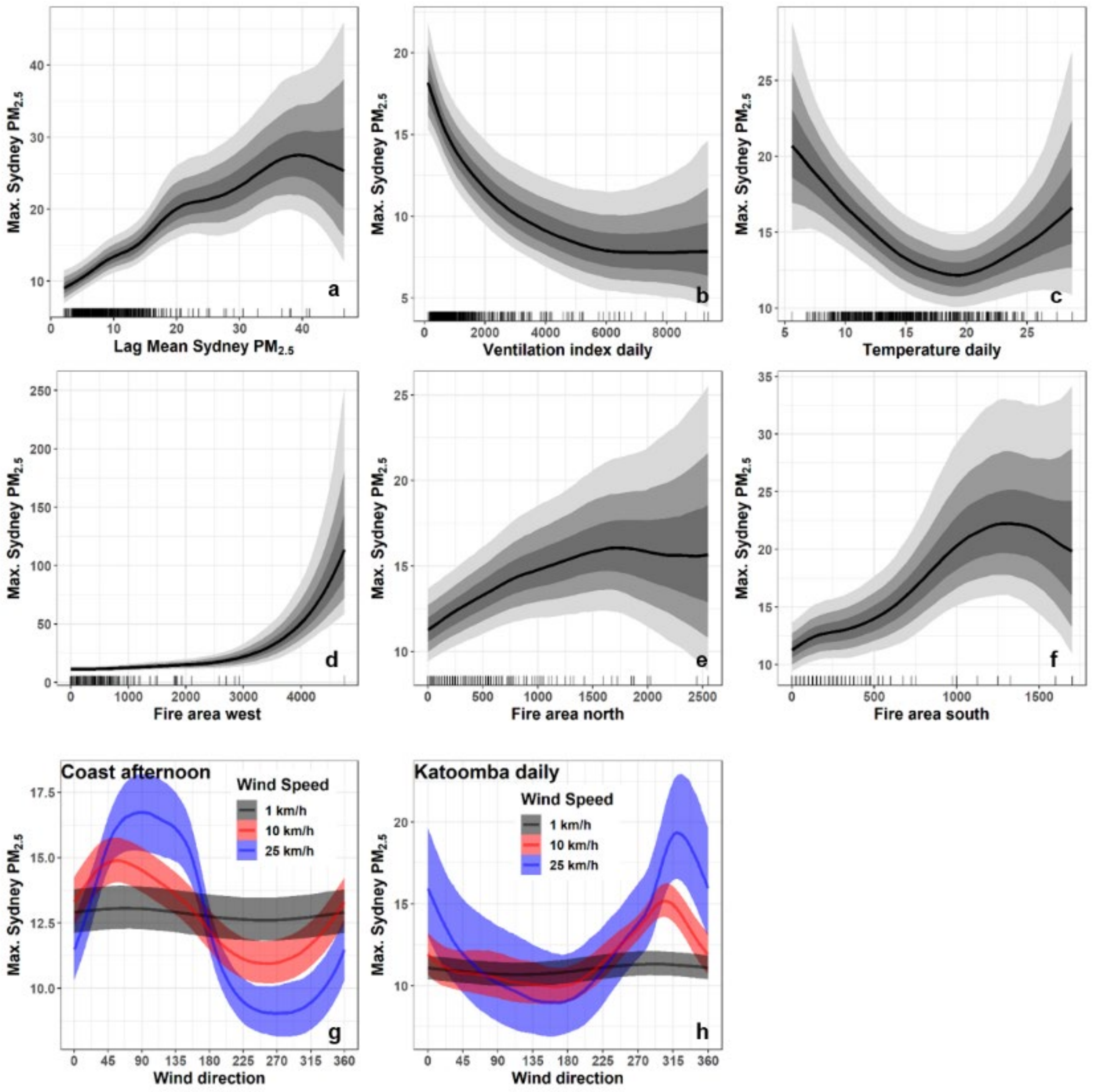


**S1 Fig A. Effects plots for maximum Sydney $PM_{2.5}$ model. The grey bands represent, from darkest to lightest grey, 0.5, 0.8 and 0.95 credible intervals. Effect of each area variable is shown with other two area variables held at zero. Other variables held at mean values. U and V wind components have been converted to wind speed and direction effects (bands show 0.5 credible interval). Wind directions are standard wind direction in degrees, i.e. 180 = southerly wind.**

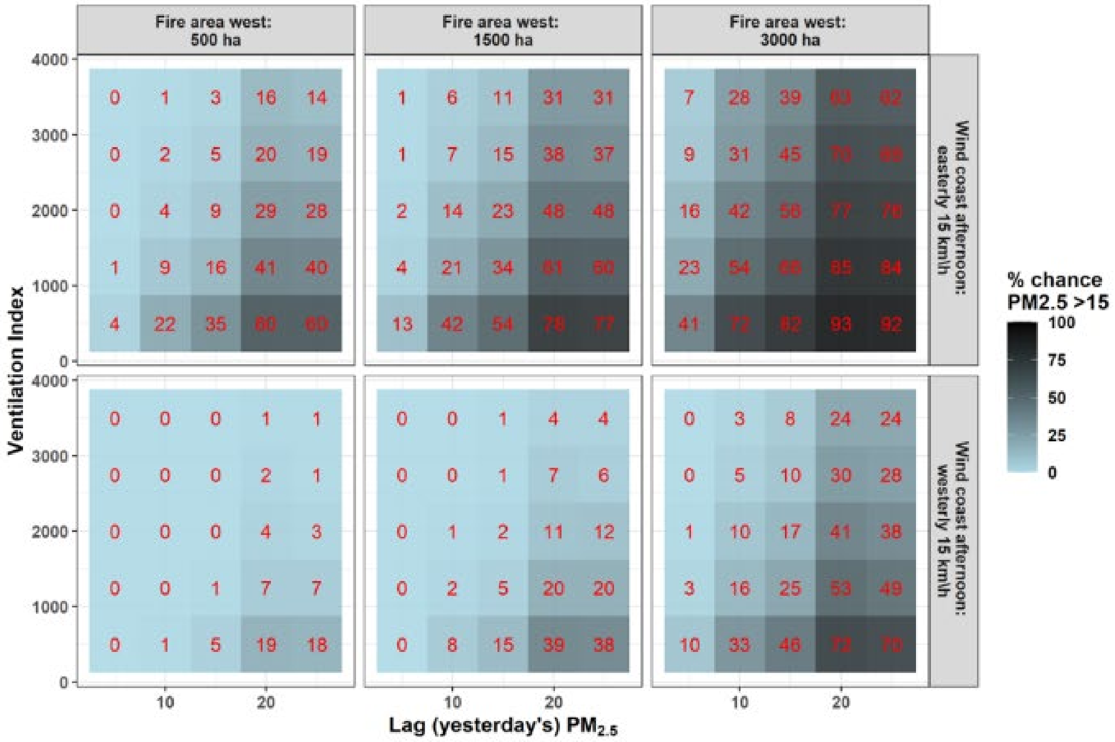


a) Mean $PM_{2.5}$ model predictions

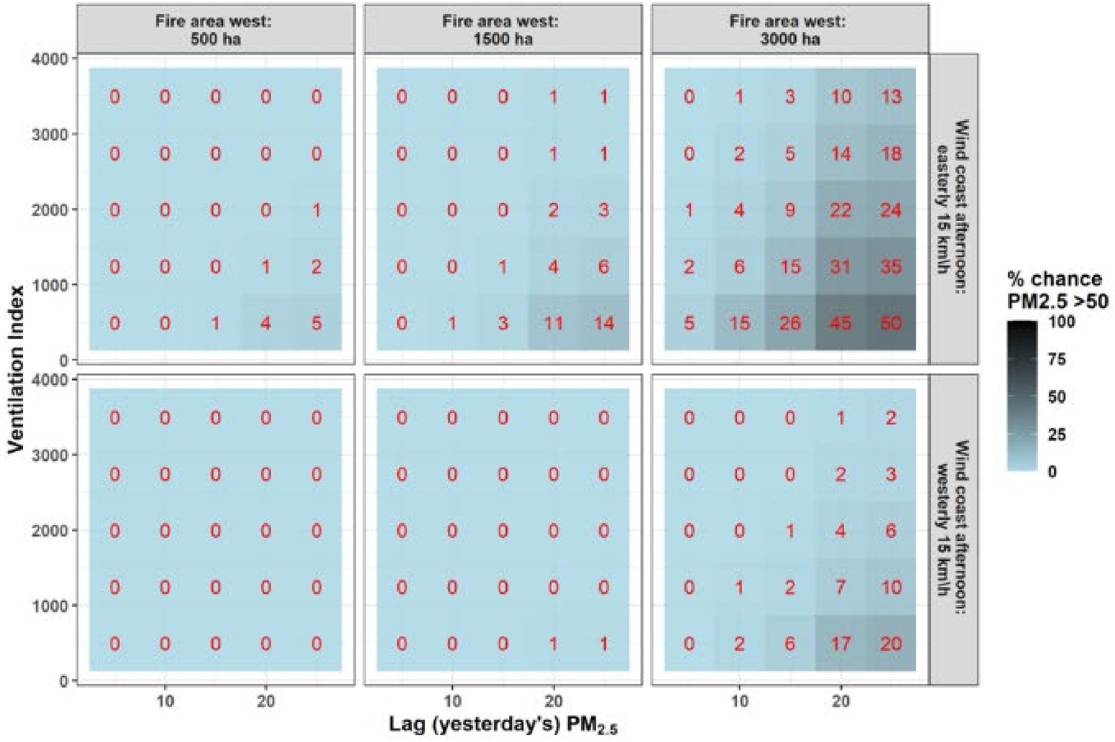


b) Maximum $PM_{2.5}$ model predictions

**S1 Fig B. Tile plot of predictions for mean (a) and maximum (b) Sydney $PM_{2.5}$ models. These are the same as in the main text, but different thresholds are used: 15 $\mu gm^{-3}$ (mean model) and 50 $\mu gm^{-3}$ (maximum model). Each coloured grid square shows the percent chance, under different predictor conditions, of exceedance above threshold (darker = higher chance, also in red text), i.e. percent of predictive distribution > threshold. Other variables held at: daily temperature 14 C, fire areas north and south = 0 ha, inland (Katoomba) wind speed and direction = 15 km $h^{-1}$ and westerly. Wind speeds and directions were calculated from the relevant U and V wind variables.**
