# Supplementary material for: Prediction of air quality in Sydney, Australia as a function of forest fire load and weather using Bayesian statistics
Source: PLoS One. 2022 Aug 24;17(8):e0272774. doi: 10.1371/journal.pone.0272774 (PMC9401134; doi:10.1371/journal.pone.0272774)
Supplement: S2 Appendix — (PDF) [file pone.0272774.s002.pdf]

S2 Appendix

This appendix details the method used to create VIIRS SNPP hotspot clusters, as mentioned in the methods section in the main text.

We employed a process to identify active fire dates from clusters of VIIRS SNPP hotspots, so those days could be included in our modelling. We used VIIRS SNPP hotspots (from here "hotspots") instead of MODIS as VIIRS are higher resolution (at nadir, 375 m vs. 1km for MODIS), thus can detect more hotspots per fire than MODIS, which reduces the chance that an active fire is missed [24]. The process to create clusters for each date for our study was to:

1. Extract all hotspots within 150 km of the Chullora AQS.
2. Remove hotspots that were not in forest by removing hotspots with low foliage projective cover score [45]. We removed hotspots with foliage projective cover < 125.
3. We also removed hotspots that were from urban industrial areas after a visual inspection indicated regular false detections, for example over the steel manufacturing and port area in Wollongong NSW (150.88 E, 34.46 S).
4. Buffer each remaining hotspot by 2.5 km and dissolve overlapping buffers into a single polygon, thus creating hotspot cluster polygons (Fig A)
5. Remove clusters that did not have at least three day or three night hotspots. This was our minimum threshold for fire activity, as we wanted to exclude very small fires such as burning heaps on farmland that can be detected by VIIRS.
6. All days with at least one cluster of hotspots identified from this process was included in our modelling

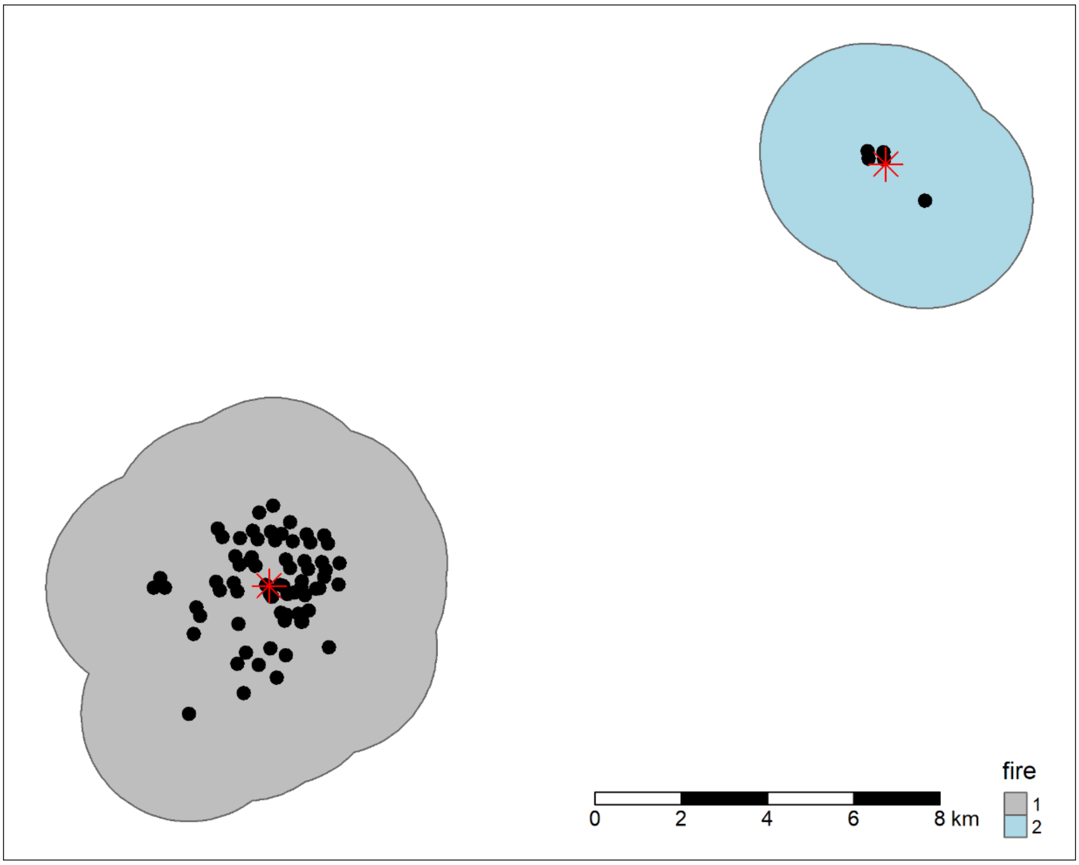

S2 Fig A. Example of creating clusters from VIIRS hotspots. Black are VIIRS SNPP hotspots, red asterisk is fire centroid, i.e. the arithmetic mean of the hotspot coordinates. The image has two separate fires. Each hotspot is buffered by 2.5 km, all overlapping buffers merged, and hotspots assigned to each separate buffer. Two separate fires are created here because of distinct fires where buffers don't overlap, i.e. greater than 2 buffer widths (5 km) apart. Fire 1 has > 50 hotspots, fire 2 has 5 hotspots. Fire area was estimated from a grid intersect method (main text), not buffer size.
