# Supplementary material for: Prediction of air quality in Sydney, Australia as a function of forest fire load and weather using Bayesian statistics
Source: PLoS One. 2022 Aug 24;17(8):e0272774. doi: 10.1371/journal.pone.0272774 (PMC9401134; doi:10.1371/journal.pone.0272774)
Supplement: Attachment — Submitted filename: Response to Reviewers.docx [file pone.0272774.s003.docx]

# Response to reviewer comments for PONE-D-22-14315

*Author responses in red italics*

*We would like to thank the authors for their efforts in reviewing our manuscript and providing insightful comments. We have made a response to each of the reviewer comments in the sections below, under the titles Reviewer #1 and Reviewer #2*

*We would like to note a change in one part of our method. We noticed an issue in our calculation of fire area for the previous manuscript. We have rectified the issue and updated the manuscript, with the results being very similar.*

*The issue was that we derived fire area from hotspots by calculating the area of each hotspot (its VIIRS pixel size) and summing the hotspot areas for each day. This led to overestimation of fire area, as hotspots acquired at different hours on the same day can overlap, meaning the same location is counted twice (or more). The over-estimation particularly affected some of the largest hazard reduction burning days.*

*In this version of the manuscript, we calculated daily area by intersecting all hotspots for a day with a 25 ha grid, and summing the area from the total number of unique intersecting grid cells.*

*We have rerun the models and updated the results and plots. The results, plots and statistics are all very similar to the previous manuscript, with the main change being higher PM2.5 is predicted at lower fire areas compared to the models in the previous version of the manuscript (See e.g. Fig 4).*

*Please see our responses to each of the reviewer comments below. Note that we have also made small changes in the manuscript (e.g. correcting spelling, grammar) that can be seen in the tracked changes version of the manuscript. We have also moved S1 and S2 appendices to separate files as required by the journal.*

Reviewer #1:

This paper has achieved its primary purpose: to develop a model to assist in scheduling HRBs around Sydney to avoid or reduce HRB-induced smoking. This model is based on Bayesian statistics and can quickly produce probabilistic predictions with given regional fire and weather variables.

Please find a few insights to consider:

Maybe you could give some details about Bayesian Statistics and GAM, not just what packages are used.

*We have added a few sentences about the advantages of these approaches and provided references, which we think is now sufficient detail. L196-206*

The predicted r2 of the model given in this paper is about 0.5. Can you provide the accuracy of some other methods like physical-based model or the empirical model? It would be better to compare the runtime and accuracy with other methods.

*We argue here that, given we present Bayesian models and their probabilistic predictions, comparison of r2 with other methods is not required. The r2 in Table 2 are for the GAMs that were only part of our model selection process, which was just a step along the way to the final models. We quote the accuracy statistics of the model-selection GAMs simply to indicate dropping MSLP and cloud cover won’t affect the models.*

*Our final models are the Bayesian models. To demonstrate accuracy/uncertainty in the models here, we show the effects plots with credible interval bounds for each variable (Fig. 4), and two ways in which the probabilistic predictions can be presented (i.e. plotting the distribution, or calculating % of predictive distribution > a threshold, Fig 5 and 6). These show uncertainty in model fitting, based on the training data, is carried through to predictions. Given this, we do not believe it is necessary to include an additional comparison of r2 via different methods.*

Some other minor things to check:

Line175: "We calculated daily mean values for all variables used in modelling, expect for the coastal wind U..." "expect" may be "except"

*Thanks for spotting this mistake, we have corrected it.*

Line218: r2 needs to be explained.

*We have added that this is the correlation-based coefficient of determination*

Reviewer #2

The manuscript discusses the development of a probabilistic model of daily PM2.5 across the Sydney area using Bayesian regression. The model provides a general, fast-to-run and operationally useful assessment of daily PM2.5 levels in the Sydney basin as a function of regional fire and weather variables. The manuscript is interesting and worth publication. It can be accepted after modification.

Detail Comment

The abstract is not very well written and represents the overall information in the manuscript. I suggest the authors improved their abstract with a clear and balance problem statement, main objective, main finding, conclusion, and suggestions.

*We have rearranged and updated the abstract in line with the reviewer’s suggestion.*

Line 28: “The model is expected….”. Another sentence that needs more concrete information based on the output of this study.

*We removed this sentence after editing the abstract*

I suggest the authors check all the references in the main text and make sure they were written based on the journal reference style. For example, in the first paragraph F. H. Johnston, 2012 and Fay H. Johnston et al., 2021 should be written as Johnston et al., 2012 and Johnston et al., 2021, respectively.

*We have fixed the name in that reference and also updated to the correct reference style and citation style (i.e. cite the reference number in square brackets).*

I would like to suggest the authors improve the flow of information and paragraphing for their introduction. Make sure each paragraph consists of one point of information.

The first few sentences about Sydney in paragraph two can be included in the methodology.

*We have made edits to the first few paragraphs to make the introduction flow better.*

*The paragraphs in the introduction now describe 1) PM2.5 2) Hazard Reduction Burning 3) Role of weather 4) Current/Physics based models 5) empirical/probabilistic approach 6) description of aims. We believe this now flows well.*

*We have moved the sentences referring to Sydney to the methodology as suggested.*

The title of the manuscript is “prediction of air quality” but is any particular reason why only PM2.5 was emphasised?

*We chose to put “air quality” in the title we thought this would be more generally understood among a wide variety of potential readers than using “PM_2.5_” in the title. PM_2.5_ is predicted by the Rural Fire Service’s current smoke prediction system because it is known to affect health and is a good surrogate for the overall pollution mixture. This means PM2.5 is the main pollution measurement that the potential operational users of our research are interested in.*

Results; “Raw Data Summary” – Descriptive Analysis Results? How about fire data? Fire data were mentioned in the “Methodology”.

*We have changed the title to “Descriptive analysis”. We have added a couple of sentences about fire area variables here. L243-246*

Section 4.2. “Model Results” seems not a suitable title for this section. I suggest the authors include a more complete title to represent the content of the section.

*We have change the title to “Model selection and predictor effects”*

Line 358-359: The authors are suggested to include their suggestions for future studies in the Conclusion. *We have now added a couple of sentences on potential future studies.*
